# Waste Clearance in the Brain

**DOI:** 10.3389/fnana.2021.665803

**Published:** 2021-07-07

**Authors:** Jasleen Kaur, Lara M. Fahmy, Esmaeil Davoodi-Bojd, Li Zhang, Guangliang Ding, Jiani Hu, Zhenggang Zhang, Michael Chopp, Quan Jiang

**Affiliations:** ^1^Department of Neurology, Henry Ford Health System, Detroit, MI, United States; ^2^Department of Physics, Oakland University, Rochester, MI, United States; ^3^Department of Psychiatry and Behavioral Neurosciences, Wayne State University, Detroit, MI, United States; ^4^Department of Radiology, Henry Ford Health System, Detroit, MI, United States; ^5^Department of Radiology, Wayne State University, Detroit, MI, United States; ^6^Department of Neurology, Wayne State University, Detroit, MI, United States

**Keywords:** cerebrospinal fluid, interstitial fluid, interstitial waste products, arachnoid villi, perineural spaces, perivascular spaces, glymphatic system, meningeal lymphatics

## Abstract

Waste clearance (WC) is an essential process for brain homeostasis, which is required for the proper and healthy functioning of all cerebrovascular and parenchymal brain cells. This review features our current understanding of brain WC, both within and external to the brain parenchyma. We describe the interplay of the blood-brain barrier (BBB), interstitial fluid (ISF), and perivascular spaces within the brain parenchyma for brain WC directly into the blood and/or cerebrospinal fluid (CSF). We also discuss the relevant role of the CSF and its exit routes in mediating WC. Recent discoveries of the glymphatic system and meningeal lymphatic vessels, and their relevance to brain WC are highlighted. Controversies related to brain WC research and potential future directions are presented.

## Introduction

Homeostasis is critical for the proper functioning of the human body, particularly, homeostasis of high-energy consuming organs like the brain. The substantial amount of toxic metabolic by-products/interstitial waste products (such as CO_2_, lactate, proteins including amyloid-β (Aβ) and tau proteins, etc.) released into the brain due to the high metabolic activity of neurons in the brain, require rapid exit by several WC mechanisms. Otherwise, the accumulation of these metabolic by-products/interstitial waste products may initiate and or exacerbate several neurological diseases, including the accumulation of Aβ in Alzheimer’s disease (AD) and tau in traumatic brain injury (TBI) (Berrouschot et al., 1998; Arbel-Ornath et al., 2013; Hinson et al., 2013; Tsitsopoulos and Marklund, 2013; Iliff et al., 2014; Kyrtsos and Baras, 2015; Plog et al., 2015; Tarasoff-Conway et al., 2015; Louveau et al., 2016; Peng et al., 2016; Xia et al., 2017; Da Mesquita et al., 2018b; Goodman et al., 2018; Reeves et al., 2020), as discussed in detail in “Waste Clearance Within the Brain Parenchyma” section. Therefore, regulating the entry and exit of various substances in the brain, as well as recycling of neurotransmitters, are essential for proper neuronal functioning and healthy aging (Harris et al., 2014; Zhao et al., 2015; Hladky and Barrand, 2016). There are two distinct systems set in place to help the brain with this type of regulation, the CSF and the vascular systems. Interestingly, the CSF and BBB are unique to the central nervous system (CNS).

**Figure 1:**
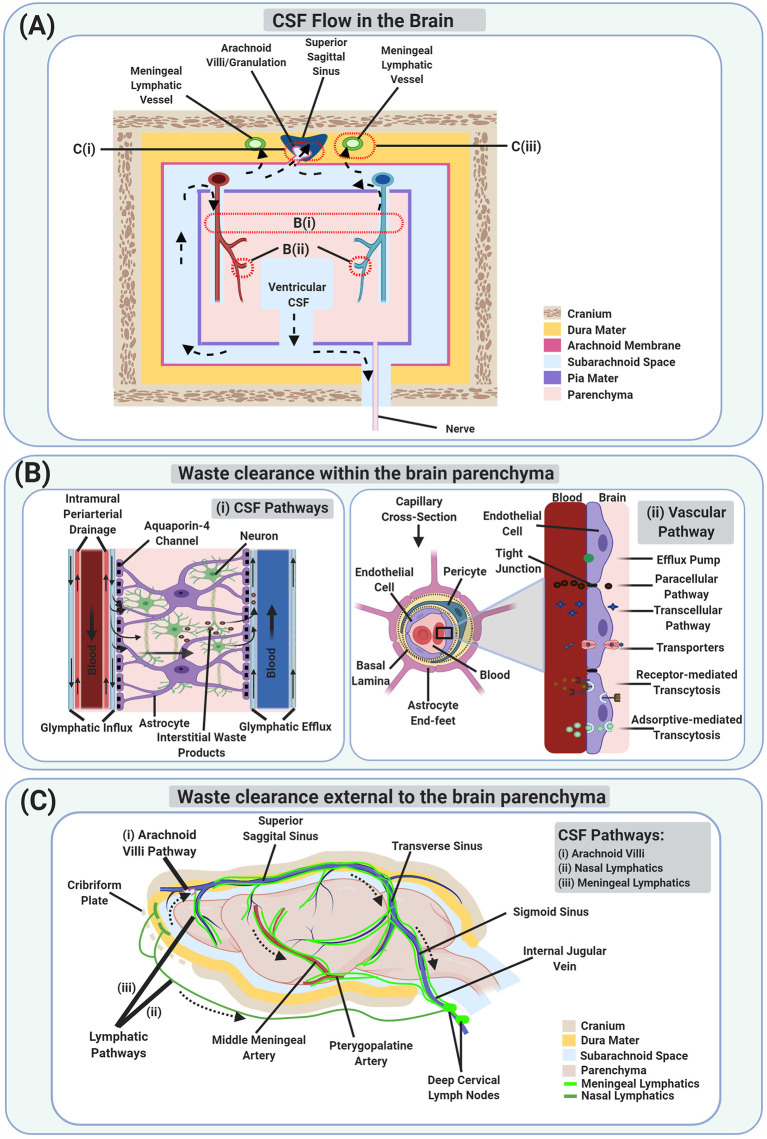
Schematic illustration of Waste Clearance (WC) in the brain. **(A)** Schematic model of cerebrospinal fluid (CSF) flow which is involved in brain WC, both within **(B)** and external to the brain parenchyma **(C)**. Dotted arrows show the movement of CSF from the ventricular compartments to the subarachnoid space, entering the brain parenchyma *via* periarterial spaces, getting mixed with interstitial waste products as well as interstitial fluid (ISF), entering the perivenous space, and then draining *via* perineural spaces and lymphatics. The waste products also reach the vascular compartments directly *via* the blood-brain barrier (BBB) and indirectly *via* CSF exiting through the arachnoid villi/granulations. **(Bi)** A magnified schematic image of the glymphatic system and intramural periarterial drainage (IPAD) pathways involved in WC within the brain parenchyma. **(Bii)** A magnified schematic image of the vascular pathway *via* the BBB at the capillary level. **(C)** A schematic image of the CSF pathways involved in brain WC *via* the arachnoid villi **(Ci)**, and lymphatic pathways [including the nasal lymphatics **(Cii)**, and the meningeal lymphatics **(Ciii)**].

The idea that CSF, which is an ultrafiltrate of the blood secreted at the choroid plexus, is involved in brain WC *via* a network of perivascular spaces has existed since at least 1974 (Cserr and Ostrach, 1974) as indicated in Figure 1A. But it did not receive much attention until 2012 when Nedergaard and colleagues expanded our understanding of the role of CSF in brain WC and proposed the glymphatic system (Iliff et al., 2012) as indicated in Figure 1Bi. They and others demonstrated that glymphatic system dysfunction is associated with a broad range of neurological diseases including, but not limited to, AD, stroke, TBI, multiple sclerosis, diabetes, and chronic traumatic encephalopathy, suggesting its involvement in virtually most neurodegenerative diseases (Iliff et al., 2012; Arbel-Ornath et al., 2013; Rangroo Thrane et al., 2013; Jessen et al., 2015; Plog et al., 2015; Louveau et al., 2016; Jiang et al., 2017; Mestre et al., 2018b, 2020a). Although dysfunction of the glymphatic system and its impact on WC from the brain is related to a variety of neurological diseases, controversial aspects concerning the convective bulk flow of CSF/ISF within the brain parenchyma and the role of aquaporin-4 (AQP4) water channels in solute transport in the glymphatic system still exist (Jin et al., 2016; Smith et al., 2017; Pizzo et al., 2018; Smith and Verkman, 2018), as discussed in detail in “The Glymphatic System” section. Augmenting the importance of the glymphatic system’s role in CNS homeostasis is the (re)discovery of the previously unappreciated meningeal lymphatic vessels in the dura (Aspelund et al., 2015; Louveau et al., 2015), originally proposed at the end of the 18th century (Lukić et al., 2003; Bucchieri et al., 2015; Da Mesquita et al., 2018a), as indicated in Figure 1Ciii. Two independent research groups in 2015 confirmed the existence of the meningeal lymphatic vessels, and furthermore, indicated that CSF tracers utilize these meningeal lymphatic vessels for brain WC.

The brain vascular WC involves the BBB as indicated in Figure 1Bii. The BBB has long been studied, particularly for its structure and role in pharmaceutical drug entry. However, its role in brain WC has been understudied. In this review, we discuss the structure of the BBB and its relationship *via* various BBB transfer mechanisms to brain WC. Until recently, the assumption has been that the BBB lacks directionality. However, recent studies have challenged this assumption, indicating that “BBB-impermeable” MRI tracers, which were thought not to cross the BBB from the blood to the brain parenchyma, may be able to cross the BBB from the brain parenchyma to the blood, suggesting directionality (Jiang et al., 2018; Hu et al., 2019). In this review, we further discuss the BBB as it pertains to brain WC.

For this review, we categorized brain WC into two levels: WC within the brain parenchyma (including the brain stroma infiltrating the brain parenchyma such as blood vessels and perivascular spaces) as indicated in Figure 1B, and WC external to the brain parenchyma as indicated in Figure 1C. The brain parenchyma consisting primarily of neurons and glial cells (surrounded by ISF) is the functional tissue for cognitive and systemic management and is considered responsible for nutrient uptake, and WC to maintain homeostasis. The remaining structural tissue in the brain, the stroma, is comprised primarily of the blood vessels, which provide the brain with oxygen and nutrients, and connective tissue. It is important to note that brain WC processes taking place within and external to the brain parenchyma in the CNS are connected, and in some instances, affect one another.

### Fluids in the Brain and Their Functions

#### CSF Production and Circulation

CSF is produced in the human brain at a rate of about 0.3–0.4 ml/min, mainly by specialized ependymal cells such as choroid plexus epithelial cells located in each of the ventricles. The CSF travels from the lateral ventricles to the third ventricle *via* the interventricular foramen of Monro and enters the fourth ventricle *via* the cerebral aqueduct of Sylvius. CSF then enters the central canal of the spinal cord *via* the obex and also reaches the subarachnoid space *via* the median aperture of Magendie and the two lateral apertures of Luschka (Johanson et al., 2008; Craven, 2010; Brinker et al., 2014; Khasawneh et al., 2018). CSF is eventually drained *via* the arachnoid villi, perineural spaces of spinal and cranial nerves, and meningeal lymphatics, as discussed in detail in “Waste Clearance External to the Brain Parenchyma” section. However, part of the CSF is also proposed to enter the brain parenchyma *via* the periarterial spaces of the penetrating arteries, mixes with interstitial waste products and ISF, enters the perivascular spaces (alongside veins by the glymphatic system or along arteries by IPAD pathway), and finally drains *via* the WC pathways, as indicated in Figure 1A, and discussed in detail in “Waste Clearance Within the Brain Parenchyma” and “Waste Clearance External to the Brain Parenchyma” section.

#### CSF Functions and Flow Behavior

The CSF has multiple functions in the brain including, providing buoyancy and buffer against injury, and by serving as the WC route, it maintains CNS homeostasis. The CSF is also very important for removing high molecular weight waste products and other debris during recovery from brain injuries, and it provides a pathway for nutrients and hormones to promote the brain’s normal development (Lehtinen et al., 2013; Hladky and Barrand, 2014).

The flow of CSF in the cerebral aqueduct, in the ventricles, and the subarachnoid space is pulsatile due to the cardiac flow (arterial hemodynamics in the choroid plexus) and pulmonary respiration (Nilsson et al., 1992; Egnor et al., 2002; Johanson et al., 2008; Brinker et al., 2014; Abbott et al., 2018; Khasawneh et al., 2018), facilitating rapid influx *via* the periarterial spaces into the brain parenchyma. However, within the brain parenchyma, CSF flow may be either *via* convective bulk flow and/or diffusion, as discussed in detail in “The Glymphatic System” section. A recent article showed that the flow of CSF in the human brain is also linked with the slow waves of neural activity and the hemodynamic fluctuations during sleep (Fultz et al., 2019).

#### ISF Production and Circulation

ISF is a filtrate of plasma secreted at the BBB of the capillary walls. ISF surrounds neural cells, makes its way through intercellular clefts of the neuropil, and is present in the extracellular spaces of the gray and white matter within the brain parenchyma. It contains interstitial waste products excreted by the cells due to cellular metabolism and nutrients from capillaries through diffusion. ISF acts as an intermediary between blood capillaries and brain cells for rapid delivery of oxygen and nutrients (such as glucose, lactate, and amino acids, etc.) for reliable cellular activity. ISF also acts as a vehicle for rapid removal of the cellular waste by-products (such as CO_2_, lactate, etc.), and cell-to-cell communication (Abbott, 2004; Howell and Gottschall, 2012; Hladky and Barrand, 2014; Lei et al., 2017; Shetty and Zanirati, 2020). The CSF act as a sink for the ISF, and the CSF-ISF exchange helps in the removal of interstitial waste products from the brain parenchyma, as discussed in detail in “Waste Clearance Within the Brain Parenchyma” section.

#### Barriers (BBB and Blood-CSF Barrier) and Their Functions

CSF and ISF are similar in composition, however, the CSF is mainly present in the cerebral ventricles and subarachnoid space covering the entire brain, whereas the ISF is extracellular fluid and surrounds the cells in the brain parenchyma. Regulation of ionic composition (such as Na^+^, Cl^−^, K^+^, Ca^2+^, Mg^2+^, etc.) and volumes of these fluids is essential for the brain cells to function effectively and is supported by barriers such as the BBB and blood-CSF barrier (Hladky and Barrand, 2014). The BBB separates blood in the vasculature from ISF and cells within the brain parenchyma and helps in maintaining the ionic composition of ISF. The BBB restricts the movement of many substances from the brain parenchyma while delivering substrates (glucose, amino acids, O_2_, etc.) for brain cell metabolism and helps in WC, as discussed in detail in “Vascular Pathway *via* the BBB” section (Hladky and Barrand, 2014, 2016).

Unlike the capillaries that form the BBB, the capillaries that supply the choroid plexuses and the circumventricular organs are fenestrated/leaky and have no tight junctions that is an essential property of the BBB (Laterra et al., 1999). However, the ependymal cells of the choroid plexuses, facing the ventricular CSF possess many microvilli consisting of a layer of cuboidal epithelial cells (having apical brush borders) overlying capillaries of the fenestrated type. The choroidal epithelial cells are connected by tight junctions and constitute the blood–CSF barrier (Cipolla, 2009; Hladky and Barrand, 2014, 2016). The blood–CSF barrier separates blood in the capillaries from the CSF, and CSF from the brain tissue and ISF, regulating the passage of molecules between them. Another type of specialized ependymal cells called tanycytes, present along the ventricular boundaries of the circumventricular organs and located around the third and fourth ventricles, also form a blood-CSF barrier (Nakada and Kwee, 2019). These specialized cells are different from other ependymal cells because they possess long processes as well as large end-feet. They connect to the fenestrated capillaries and neurons in the hypothalamic parenchyma utilizing their long processes, prevent the diffusion of molecules from the blood into the CSF utilizing their tight junctions, and maintain the brain homeostasis (Weindl and Joynt, 1972; Mullier et al., 2010; Langlet et al., 2013; Hladky and Barrand, 2014; Abbott et al., 2018).

## Waste Clearance Within the Brain Parenchyma

Most neurodegenerative diseases are related to the improper accumulation of cellular waste by-products. Among them, misfolded proteins are the most difficult to clear from the brain, and their build-up leads to diseases such as AD (Nedergaard, 2013). Although chaperone proteins in brain cells help proteins fold correctly and limit their aggregation, folding and aggregation errors do occur, and some proteins are more vulnerable than others. If misfolded proteins are not identified and removed from the brain parenchyma, then these interstitial waste products can cause or exacerbate neurological diseases (Powers et al., 2009; Hartl et al., 2011; Wolfe, 2012). Aβ and tau proteins are the two most concerning neurotoxic waste proteins due to their accumulation in the brain and association with neurological diseases such as AD and TBI (Berrouschot et al., 1998; Arbel-Ornath et al., 2013; Hinson et al., 2013; Tsitsopoulos and Marklund, 2013; Iliff et al., 2014; Kyrtsos and Baras, 2015; Plog et al., 2015; Tarasoff-Conway et al., 2015; Louveau et al., 2016; Peng et al., 2016; Xia et al., 2017; Da Mesquita et al., 2018b; Goodman et al., 2018; Reeves et al., 2020). Aβ is a small protein fragment that is cleaved from a larger amyloid precursor protein, which extends from the inside to the outside of brain cells. Aβ eventually forms amyloid-plaques in the extracellular spaces of the brain parenchyma, found in AD patients (Murphy and LeVine, 2010; Haass et al., 2012). These Aβ aggregates impair communication between neuronal cells, cause nerve inflammation, and are a common cause of dementia in the elderly. In transgenic mouse models of AD, intracellular Aβ has been associated with cognitive deficits, tau-phosphorylation, synaptic dysfunction, and neuronal death (LaFerla et al., 2007).

Tau protein is an intracellular protein that mainly exists in neurons and helps stabilize microtubules. They are released to the extracellular space after a rapid increase in neuronal activity (Yamada et al., 2014). When misfolded and hyperphosphorylated, tau proteins create neurofibrillary tangles which disrupt neuronal signal transmission, cause microtubules to disassemble, and promote mitochondrial dysfunction. In addition to Aβ protein, tau protein is also one of the main features of AD (Gong and Iqbal, 2008; Mandelkow and Mandelkow, 2012). In a recent study, it has been suggested that both extracellular Aβ plaques and intracellular tau neurofibrillary tangles work together in synergy in generating AD (Busche and Hyman, 2020). The accumulation and movement of tau protein in the extracellular space have been related to many neurodegenerative diseases, such as chronic traumatic encephalopathy, Parkinson”s disease, prion disease, etc. (Frost and Diamond, 2010; Jucker and Walker, 2011; Iliff et al., 2014; Pirscoveanu et al., 2017; Edwards et al., 2020). Since the Aβ and tau accumulation impairs neurons, they need constant maintenance and repair, which are essential for learning, memory, and neurological function.

In some instances, the waste products of certain cells can be the fuel for other types of cells. For a long time, the metabolic waste product “lactic acid” released by astrocytes in the brain was regarded as a waste product, however, it has been shown to be an important fuel for many cells (including neurons; Proia et al., 2016). In the brain, lactic acid is absorbed by neurons as fuel and used to synthesize acetyl-CoA for the tricarboxylic acid cycle. It also acts as a signal molecule in the brain, participates in memory formation, and provides neuroprotection (Gladden, 2004; Berthet et al., 2012; Proia et al., 2016).

Also, in the CNS, neurotransmitters (such as acetylcholine, dopamine, glutamate, serotonin, norepinephrine, etc.) play a key role in the transmission of information in chemical synapses, and even small changes in their concentrations may lead to neurodegenerative diseases (Harris et al., 2014). So, the release and recycling of neurotransmitters in the brain parenchyma are very important for the normal function of neurons. As an action potential propagates to the terminal of a presynaptic neuron, vesicles storing the neurotransmitter fuse with the cell membrane and release the neurotransmitter into the synapse. Once the neurotransmitter is secreted into the synaptic cleft, it binds to specific receptors on the postsynaptic cell, thereby generating a postsynaptic electrical signal, initiating rapid communication between neurons. Then the transmitter must be cleared quickly or chemically inactivated so that the postsynaptic cell can participate in another cycle of neurotransmitter release, binding, and signaling.

Neurotransmitter clearance mechanisms include diffusion, enzymatic degradation, reuptake by presynaptic nerve terminals, uptake by glial cells through transporters located primarily on the surface of glial cells (Rothstein et al., 1996; Purves et al., 2001; Chaturvedi et al., 2014; Harris et al., 2014; Gross and von Gersdorff, 2016). If these mechanisms are deregulated, the dysfunctional neurotransmitter signaling can negatively alter brain function such as the increased release of presynaptic neurotransmitter, direct release of neurotransmitter from glial cells, impaired ability to clear neurotransmitter from the extracellular space which may lead to excessive stimulation of neurotransmitter receptors, resulting in neurotoxicity, and dysfunctional neurotransmitter signaling is implicated in various diseases (Caudle et al., 2007; Bisaglia et al., 2013; Paula-Lima et al., 2013; Harris et al., 2014).

The brain parenchyma (which consists of the neurons and glial cells) is infiltrated by the brain stroma (which contains the blood vessels and perivascular spaces) and lacks lymphatic vessels. WC at this level consists of the interaction of the brain parenchyma with the blood vessels and perivascular spaces, which eventually help transport the waste products outside the cranium. Therefore, waste products can only be removed from the brain parenchyma by: (a) CSF pathways *via* the perivascular (as indicated in Figure 1Bi) and periaxonal/perineural routes; (b) the vascular pathway *via* the BBB as indicated in Figure 1Bii. Waste products which are small in size (such as CO_2_, K^+^, lactate, etc.) exit *via* the BBB. Whereas, waste products which are larger in size, polar in nature (such as sucrose, inulin, albumin, dextran, etc.), and lack specific transporters to cross the BBB, exit *via* perivascular or periaxonal/perineural routes. However, special solutes which are larger in size, such as Aβ (Shibata et al., 2000; Bell et al., 2007; Qosa et al., 2014), insulin (Duffy and Pardridge, 1987), and transferrin (Banks et al., 1988), also exit *via* the BBB with the help of transcytosis. Yet other substances are first metabolized to other products (such as glucose metabolizing to CO_2_ and water) before exiting. However, Na^+^, Cl^−^, and water are transported by both vascular and CSF pathways (Hladky and Barrand, 2018).

### CSF Pathways *via* the Perivascular and Periaxonal/Perineural Spaces

The idea that CSF infiltrates the brain parenchyma and is involved in WC has been around for a long time. There is an agreement in the field regarding the utility of the perivascular spaces in CSF-related WC. However, the exact pathway undertaken by CSF-related WC inside the brain parenchyma, as pertaining to the perivascular spaces, is very much under debate. Thus far, two pathways have been strongly put forward: the intramural periarterial drainage (IPAD) and the glymphatic system. These pathways are discussed at length in this section. There appears to be consensus in the field regarding the utility of the periaxonal/perineural spaces in CSF-related WC (which span brain WC within and external to the brain parenchyma).

#### Perivascular Spaces

By looking at the distribution of dye “blue dextran 2000KDa” (Cserr and Ostrach, 1974) and “horseradish peroxidase” injected into the caudate nucleus of rats, Cserr et al. (1977) demonstrated that the interstitial fluid (ISF) interacts with and drains into the CSF *via* bulk flow along the cerebral blood vessels (i.e., the perivascular spaces). The convective bulk flow transport of tracers was also observed in the periventricular regions and between the fiber tracts in the white matter (Cserr et al., 1977). Subsequent experiments demonstrated similar efflux rates of various radiolabeled tracers from the brain ISF, despite big differences in their molecular size and diffusion coefficients, implying the convective bulk flow nature of clearance from the brain parenchyma (Cserr et al., 1977, 1981). Moreover, microinjection of radio-iodinated albumin into the caudate nucleus, internal capsule, or midbrain suggests ISF drainage out of the brain parenchyma along blood vasculature (Szentistvanyi et al., 1984).

Further insights by Rennels et al. (1985, 1990) suggest that CSF may enter into the brain parenchyma *via* perivascular spaces, and they demonstrated a perivascular distribution of horseradish peroxidase (HRP) when injected into the subarachnoid space of cats and dogs. HRP tracer was present in the perivascular spaces along large penetrating arteries, into the basal lamina of capillaries, venules, and perivascular spaces of veins. The HRP reached extracellular spaces (ECS) throughout the CNS *via* paravascular pathways (i.e., CSF fluid pathways) present along intraparenchymal vasculature in ~10 min; suggesting the exchange of solutes in CSF with the ECS by a convective bulk flow supported by cerebral arterial pulsations rather than by diffusion alone (Rennels et al., 1985, 1990).

##### Intramural Periarterial Drainage (IPAD)

A study in 2008, in which various soluble tracers were injected into the corpus striatum of mice, demonstrated diffusion of tracers through the extracellular spaces of the brain parenchyma and then convective bulk flow drainage along the basement membrane of capillaries and arterial walls. This indicated that ISF and waste products drain *via* the same pathway, termed IPAD (Carare et al., 2008); joining the CSF in the subarachnoid space or dissipation at the base of the skull for further drainage into the cervical lymph nodes (CLNs) and/or blood. A recent study in mice demonstrated that following injection of fluorescent Aβ into the cisterna magna, the CSF tracer enters the brain parenchyma along pial-glial basement membranes. It then mixes with ISF and interstitial waste products and exits along the basement membranes in the walls of capillaries and the basement membranes between smooth muscle cells in the walls of the cortical arteries (IPAD pathway; Albargothy et al., 2018) as indicated in Figure 1Bi. Other studies using mathematical and computational models suggest that arterial pulsations, which were initially considered as a motive force for IPAD, cannot be the only driving force for the IPAD pathway to efficiently account for the experimental observations (Diem et al., 2017); however, vasomotion induced by contraction and relaxation of cerebrovascular smooth muscle cells are suggested to be a more plausible driver for IPAD (Aldea et al., 2019).

##### The Glymphatic System

In 2012, Nedergaard and colleagues performed a series of fluorescence microscopy experiments that demonstrated the path of CSF circulation within the brain parenchyma *via* a network of perivascular spaces, termed the glymphatic system. The glymphatic system hypothesis describes subarachnoid CSF entry into the brain parenchyma through the periarterial spaces, mixing with parenchymal ISF and interstitial waste products facilitated by AQP4 water channels embedded in astrocytic end-feet, and drainage through perivenous spaces surrounding the veins (Iliff et al., 2012, 2013a; Nedergaard, 2013; Rangroo Thrane et al., 2013; Xie et al., 2013; Plog et al., 2015; Nedergaard and Goldman, 2016; Benveniste et al., 2017b) as indicated in Figure 1Bi. So whereas the IPAD pathway demonstrated CSF brain parenchymal circulation through the separate periarterial basement membranes (convective influx along pial-glial basement membranes as suggested in the glymphatic pathway and the efflux along basement membranes between smooth muscle cells in the walls of arteries (Albargothy et al., 2018), the glymphatic system pathway demonstrated CSF circulation *via* the perivascular spaces surrounding the arteries and veins.

Specifically, Nedergaard and colleagues injected tracers into the cisterna magna of mice and two-photon imaging studies of these mice indicated that the tracer size matters when determining brain parenchymal tracer distribution (Iliff et al., 2012). Small molecular weight CSF fluorescent tracer [Texas Red–dextran-3 (TR-d3): molecular size, 3 kD] injected into mouse cisterna magna was observed within periarterial spaces and within brain parenchyma within 30 min; indicating unrestricted movement of small molecular weight tracers into the interstitium. However, large molecular weight CSF fluorescent tracer [fluorescein isothiocyanate-dextran-2000 (FITC-d2000): molecular size, 2,000 kD] predominantly remained confined in the periarterial spaces; highlighting the role of the 20 nm-wide astrocytic end-feet gap junctions (Iliff et al., 2012). The observation that both small and large molecular weight tracers entered perivascular spaces alongside arteries indicates that the transport is facilitated by CSF convective bulk flow *via* perivascular spaces.

Transport by AQP4 water channels is thought to play a dominant role in mediating brain parenchymal entry of CSF tracer from the periarterial spaces (Iliff et al., 2012; Mestre et al., 2018a), which is driven by the hydrostatic pressure generated by arterial pulsations as proposed in the glymphatic system (Iliff et al., 2013b). Cerebral arterial pulsations are said to drive the CSF convective bulk flow in the perivascular spaces and are the key contributors of CSF entry into the brain parenchyma allowing CSF-ISF fluid exchange in the glymphatic system (Iliff et al., 2013b; Mestre et al., 2018b). Additionally, astroglial AQP4 support the convective bulk flow of ISF through the brain parenchyma to drive the interstitial waste products (Aβ) to the perivenous spaces; in AQP4-null mice, the interstitial waste products clearance is reduced by nearly 70% (demonstrated using intraparenchymal injections in mice; Iliff et al., 2012).

The CSF mixed with ISF and interstitial waste products such as Aβ is drained primarily through the perivenous spaces, which may then reenter associated CSF compartments, either within the ventricles (for internal cerebral veins), or in the subarachnoid space (for caudal rhinal veins) (Iliff et al., 2012), or may drain to the blood across the vasculature *via* specific transport mechanisms at the BBB (Iliff et al., 2012; Ueno et al., 2016; Hladky and Barrand, 2018; Jiang et al., 2018; Hu et al., 2019). The waste products, which reach the CSF in subarachnoid space may then exit the cranium either *via* arachnoid granulations or meningeal lymphatic vessels or along cranial and spinal nerves (Ueno et al., 2016; Louveau et al., 2017; Hladky and Barrand, 2018) as indicated in Figure 1A, and discussed in “Waste Clearance External to the Brain Parenchyma”. However, and as an attempt to reconcile the IPAD and the glymphatic system pathways, some speculate that the CSF-ISF mixture may recirculate in the brain parenchyma *via* periarterial spaces and accumulate within the basement membranes of smooth muscle cells, triggering neurological conditions (Iliff et al., 2012).

The glymphatic system impacts the physiology of the body, such as sleep (Nedergaard and Goldman, 2016), body posture (Lee et al., 2015), blood pressure (Kyrtsos and Baras, 2015), aging (Zieman et al., 2005; Chen et al., 2009; Fleischman et al., 2012; Iliff et al., 2013b; Kress et al., 2014; Jessen et al., 2015; Kyrtsos and Baras, 2015; Da Mesquita et al., 2018b; Zhou et al., 2020), anesthesia (Benveniste et al., 2017a), etc. Slight changes in normal physiology can affect the clearance rate of interstitial waste products from the brain. The glymphatic system helps in the removal of Aβ, a protein which is a trademark of AD, from the brain (Iliff et al., 2012; Tarasoff-Conway et al., 2015; Simon and Iliff, 2016). Decreased glymphatic clearance and increased Aβ accumulation have been seen in the aged brain (Kress et al., 2014), sleep deprivation (Xie et al., 2013), depression (Xia et al., 2017), and obstructive sleep apnea (Ju et al., 2016). Whereas, voluntary exercise (He et al., 2017) and Omega-3 polyunsaturated fatty acids (Ren et al., 2017) promote the glymphatic clearance of Aβ. Dysfunction/impairment of the glymphatic system has been associated with several neurological diseases including AD (Zarow et al., 2003; Peng et al., 2016; Reeves et al., 2020), TBI (Mondello et al., 2011; Morris et al., 2011; Magnoni et al., 2012; Tsitsopoulos and Marklund, 2013; Iliff et al., 2014; Jessen et al., 2015; Plog et al., 2015), small vessel disease (SVD) (Charidimou et al., 2016; Mestre et al., 2017), stroke (Gaberel et al., 2014; Goulay et al., 2017; Mestre et al., 2020a), diabetes (Jiang et al., 2017), microinfarcts (Venkat et al., 2017; Wang et al., 2017), migraines (Schain et al., 2017), glaucoma (Wostyn et al., 2017), etc. In most of the studies relating glymphatic system impairments to neurological diseases, it is not clear if the impairments are the cause or the result of these diseases, and therefore the coupling of glymphatic system impairment and neurological diseases requires further investigation (Mestre et al., 2017).

Various modeling techniques such as multi-compartment kinetic models (Lee et al., 2015; Davoodi-Bojd et al., 2019), optimal mass transport models using Eulerian (Ratner et al., 2015, 2017), and Lagrangian (Elkin et al., 2018, 2020) approaches have been implemented using data from MRI to visualize the dynamics of the glymphatic system. Lee et al. (2015) utilized a two-compartment model based on contrast-enhanced MRI data of CSF (Gd-DTPA) tracer and suggested that glymphatic system transport is most efficient in rats sleeping in the lateral position as compared to prone and supine positions. The limitation of this model includes the use of a global input function that cannot provide accurate measurements for local regions, respectively (Davoodi-Bojd et al., 2019). Davoodi-Bojd et al. (2019) by utilizing the local input function for each region, employed the multi-compartment model to quantitatively map the glymphatic system pathway and derived associated parameters. This model also suggested the reduced clearance in the diabetes mellitus rats, in agreement with the previous findings (Jiang et al., 2017). The limitation of this model lies in the absence of a diffusion term, which may cause errors in WC within the brain parenchyma.

Ratner et al. (2015, (2017) used the optimal mass transport algorithm with the Eulerian approach to identify the fluid flow patterns in the glymphatic transport and to ascertain its function in normal and therapeutic conditions. The optimal mass transport technique provided a good estimate of the glymphatic flow directionality; however, it did not provide a quantitative evaluation of glymphatic transport as well as efflux pathways out of the brain. Later, Elkin et al. (2018, 2020) measured the glymphatic transport using the optimal mass transport algorithm with the Lagrangian approach instead of the Eulerian approach. Their model based on an adjusted continuity equation was able to distinguish the glymphatic flow patterns in rats under two different anesthetics. The main advantage of this model is that compared with the traditional optimal mass transport model, it not only reduced the error by five times, but at the same time, it provides qualitative information about fluid flow, rather than quantitative information. Eulerian and Lagrangian methods differ in describing the fluid motion and its associated properties. The Eulerian approach identifies the fixed location in a flow field and tracks the properties (such as temperature, concentration, etc.) at the same location as the fluid passes with time. However, the Lagrangian approach identifies the particles in the fluid and tracks them as they move with time along with their properties (such as velocity, temperature, density, mass, concentration, etc.). The Lagrangian approach is advantageous over the Eulerian approach to envision the time-varying trajectories of particles in a single visualization (Elkin et al., 2020).

Various studies suggested the presence of the glymphatic system in the human brain consistent with the animal studies (Eidsvaag et al., 2017; Ringstad et al., 2017; Eide et al., 2018; Ringstad et al., 2018; Eide and Ringstad, 2019a, b; Watts et al., 2019). Ringstad et al. (2017) pioneered the use of MRI to study the glymphatic system in patients with idiopathic normal pressure hydrocephalus (iNPH) and found a delay in the clearance of CSF tracer (gadobutrol) from the subarachnoid space and brain parenchyma compared to the healthy reference group. They also suggested the increase in glymphatic system clearance during sleep. Eide et al. (2018) demonstrated *in vivo* CSF tracer drainage to the cervical lymph nodes in humans at the same time corresponding to the peak CSF tracer uptake by the glymphatic system. Ringstad et al. (2018) examined the brain-wide glymphatic enhancement of CSF tracer (gadobutrol) using MRI for time points up to 4 weeks and showed delayed clearance in iNPH patients compared to healthy individuals. CSF tracer uptake in the human brain was slower compared to the rodent brain which may be due to physiological differences between human and rodent brain such as difference in vascular pulsatility, metabolic rate, brain mass, AQP4 density, et cetera (Benveniste et al., 2019). Watts et al. (2019) demonstrated a quantitative glymphatic flow in a man utilizing MRI imaging before and after intrathecal injection of CSF tracer (gadobutrol). Also, based on MRI data in iNPH patients, it has been proposed that reduced CSF clearance in the entorhinal cortex and white matter is associated with the impairment of the glymphatic system, which might be the cause of dementia in iNPH (Eide and Ringstad, 2019a, b). A recent human study utilized MRI to identify the clearance of MRI contrast agent (gadodiamide) in the glymphatic system and the existence of putative meningeal lymphatic vessels and suggested the impairment of both pathways with aging (Zhou et al., 2020), consistent with the animal studies.

There are some controversial aspects related to the glymphatic system hypothesis primarily based on: (a) convective bulk flow within the brain parenchyma; and (b) AQP4 dependent solute transport within the brain parenchyma. (a) The glymphatic system proposed the size-independent convective bulk flow of solutes within the brain parenchyma. Some experiments in the past also demonstrated the similar convective bulk flow nature of solute clearance from the brain parenchyma independent of their molecular size (Cserr et al., 1977, 1981; Rennels et al., 1985, 1990). However, Smith et al. (2017) recently demonstrated a diffusive transport in the brain parenchyma dependent on the solute size, consistent with their diffusion coefficients. Moreover, some modeling studies showed the movement of tracer through extracellular spaces in the brain parenchyma primarily by diffusion rather than a convective bulk flow, inconsistent with the glymphatic hypothesis (Jin et al., 2016; Holter et al., 2017). Similarly, numerous studies in the past utilizing a variety of tracers have also concluded the diffusion-based solute movement within the brain parenchyma (Rall et al., 1962; Brightman and Reese, 1969; Levin et al., 1970; Patlak and Fenstermacher, 1975; Tao and Nicholson, 1996; Brightman, 2002). In addition, early and more recent studies indicate that high hydraulic resistance from tight (38–64 nm) and tortuous extracellular spaces cannot allow bulk flow even if there is a substantial pressure difference between the periarterial and perivenous spaces to drive the convective bulk flow (Fenstermacher and Patlak, 1976; Thorne and Nicholson, 2006; Sykova and Nicholson, 2008; Verkman, 2013; Wolak and Thorne, 2013; Holter et al., 2017; Pizzo and Thorne, 2017; Pizzo et al., 2018).

However, more recent articles (Ray et al., 2019; Thomas, 2019; Mestre et al., 2020b) and a new model utilizing the Lagrangian approach for optimal mass transport reveals that convective bulk flow is the dominant transport mechanism in the perivascular spaces; while both, convective bulk flow and diffusion, are important mechanisms for transport within the brain parenchyma (Koundal et al., 2020). Based on the available evidence, it is also likely that CSF enters into the periarterial spaces of large vessels *via* convective bulk flow, enters the brain parenchyma, and mixes with ISF and waste products *via* diffusion at the level of capillary/microvessels as suggested by several groups (Smith et al., 2015; Asgari et al., 2016; Jin et al., 2016; Holter et al., 2017; Smith et al., 2017; Hannocks et al., 2018; Pizzo et al., 2018), with a possibility of pericapillary convective bulk flow along with diffusion in the basal lamina to connect to the perivascular spaces of arterioles and venules and finally drains *via* perivenous spaces (Abbott et al., 2018). However, the debate remains whether the convective bulk flow is restricted only to the perivascular spaces or occurs within the brain parenchyma along with the diffusion.

(b) The role of AQP4 water channels in convective glymphatic transport has been questioned. Smith et al. (2017) demonstrated that in AQP4 null mice, the transfer of fluorescent solutes from CSF into the brain parenchyma was not impaired compared to wild-type mice; inconsistent with glymphatic studies. Later, these results were investigated by five different lab groups in AQP4 knockout mice and it was demonstrated that influx of CSF tracer into the brain parenchyma was significantly impaired compared to wild-type controls; suggesting CSF influx and ISF efflux are highly dependent on AQP4 water channels (Mestre et al., 2018a).

The constant flow injection technique used by Iliff et al. (2012) in glymphatic studies has also been questioned because of its possibility to cause an increase in the intracranial pressure and contribution to parenchymal tracer distribution (Smith and Verkman, 2018). However, Mestre et al. (2020b) suggested that by the time CSF tracer enters into the brain parenchyma, intracranial pressure already returns to baseline. Also, it has been shown that even a six-fold difference in CSF tracer infusion speed does not influence the mean CSF speed in the perivascular spaces (Bedussi et al., 2018; Mestre et al., 2018b, 2020b).

The inconsistencies in the results of the glymphatic system between two research groups Smith et al. (2017) and Iliff et al. (2012) are suggested to be due to some technical differences in their experimental procedures including, the intraparenchymal injections, choice of anesthesia, and mice of different ages.

Smith et al., 2017 inserted a beveled glass micropipette ~20 mm in diameter into the cisterna magna as well as striatum for pulsed pressure injections, whereas (Iliff et al., 2012) used a 30–33 gauge metal syringe for constant flow rate injections into the cisterna magna and brain parenchyma. A recent study demonstrated that the surgical procedure associated with the glass micropipette for injection into the striatum or cortex as used by Smith et al. (2017) acutely suppressed CSF tracer influx into the brain parenchyma, suggesting that this type of procedure is traumatic and should be avoided for glymphatic studies (Mestre et al., 2018a). Concerning this, another study in the past showed that the cannula insertion into the striatum of the rats and the rate of drug infusion significantly affect the convective distribution of molecules in the process of convection-enhanced delivery (CED) of therapeutic drugs; also suggesting a tissue distortion caused by cannula (Chen et al., 1999). Another CED study showed that due to a slight difference (~1 mm) between the cannula placement in the two sites of the rat’s ventral hippocampus, the distribution pattern of the tracer was different. When the cannula was inserted into the hippocampus CA1 subfield, the infusate occupied the hippocampus, dentate gyrus, and CSF regions (ventricles, velum interpositum, and midbrain cisterns). However, when the cannula was inserted between the alveus and the corpus callosum, the preferred distribution along the direction of axonal fiber tracts of the white matter was determined. The tracer occupied the alveus and fimbria of the hippocampus, corpus callosum, and minute amounts reached into the CSF space (Kim et al., 2012).

Also, Smith et al., 2017 used avertin as an anesthetic agent compared to ketamine/xylazine used by Iliff et al. (2012). A recent article by Hablitz et al. showed that anesthetic choice by Smith et al. (2017) may have caused the discrepancy in results. They tested glymphatic CSF tracer influx in mice using six different anesthetic agents and found that glymphatic influx was highest under ketamine/xylazine anesthesia, followed by isoflurane along with dexmedetomidine and then pentobarbital. Anesthetic agents α-chloralose, avertin and, isoflurane exhibited low glymphatic tracer influx (Hablitz et al., 2019).

Another difference is that used mice of either sex and 3–6 months of age whereas Iliff et al. used male mice 8–12 weeks of age. Since the CSF-ISF exchange dramatically decreases with age (Kress et al., 2014), it has been suggested by Mestre et al. (2020b) that it is crucial to compare the mice within the narrow age range as done by Iliff et al. (2012).

Besides that, recent studies using *in vivo* observation of periarterial space geometry demonstrated that the periarterial spaces are not actually in the shape of circular annula as represented in most of the mathematical models; instead, the outer boundaries of periarterial spaces are oblate and should best be represented as circles surrounded by ellipses, which have much less hydraulic resistance by several folds (Tithof et al., 2019). Our previous review article provides information on various MRI studies related to the glymphatic system, modeling techniques used to evaluate the glymphatic flow pathways, the association of normal physiology and neurological diseases with the glymphatic system, and deficiencies in the current understanding of the glymphatic system (Kaur et al., 2020).

#### Periaxonal/Perineural Spaces

Various studies have indicated that there is a fast efflux of ISF and waste products along white matter/axon tracts, in addition to the perivascular spaces discussed above (Cserr et al., 1977; Rosenberg et al., 1980; Abbott, 2004; Sykova and Nicholson, 2008; Brinker et al., 2014). As a gross extension of this, perineural spaces along all twelve pairs of cranial nerves are also involved in CSF-related WC; particularly the olfactory (CNI) and optic (CNII) cranial nerves, as they are anatomically and histologically considered as white matter tract extensions (Mabray et al., 2015). All the cranial nerves’ nuclei/cell bodies lie within the brain parenchyma. CNI and CNII emerge/originate from the cerebrum (CNI from the olfactory bulb and CNII from the lateral geniculate nuclei), and the rest of the cranial nerves (III-XII) have their cell bodies in the brainstem. After emerging, cranial nerves travel within the skull and then exit *via* foramina in the skull to reach their final destinations (Mabray et al., 2015; Krainik and Casselman, 2020). Therefore, perineural spaces accompanying the cranial nerves start within the brain parenchyma and make their way outside the brain parenchyma, along the cranial nerves as indicated in Figure 1A. The role of the perineural spaces in CSF-related WC is further discussed in the next section (WC external to the brain parenchyma).

### Vascular Pathway *via* the BBB

The BBB consists of continuous endothelial cells joined together with tight junctions which are further supported by pericytes, the basement membrane, and the astrocytic endfeet. The microvascular network in the brain is composed of arterioles, venules, and capillaries. They all display the BBB properties, however, capillaries represent the largest microvasculature with the tightest junctions at BBB as compared to arterioles and venules (Wilhelm et al., 2016; Sweeney et al., 2019). The BBB makes the vascular pathway highly selective, as compared to the CSF pathways, due to the presence of tight junctions in the gaps between endothelial cells as indicated in Figure 1Bii. The effective exchange of products between the brain parenchyma and blood is very important for the influx of various nutrients and the efflux of metabolic waste products-critical for proper neuronal functioning. Glucose, CO_2_, O_2_, water, and amino acids are some of the major substances that must be rapidly transported in and out of the brain parenchyma in large quantities to maintain brain homeostasis. Mechanisms of BBB transfer (Hladky and Barrand, 2016, 2018) include:

#### Passive, Non-specific Transport

A passive, non-specific transfer can occur *via* a paracellular or transcellular pathway. Due to the presence of tight junctions at the BBB, the paracellular pathway is highly restricted compared to the transcellular pathway; however, this pathway is still permeable to small solutes. Most of the passive, non-specific transfer occurs *via* a transcellular pathway in which waste products such as water, ethanol, methanol, glycerol, urea, isopropanol, ethylene glycol, etc. must pass/diffuse through the endothelial cell, crossing both the luminal and abluminal membranes (Hladky and Barrand, 2018). Yet other non-specific substances such as “BBB-impermeable” MRI tracers which classically cannot cross the BBB from the blood to the brain parenchyma, may be able to cross the BBB from the brain parenchyma to the blood; suggesting BBB directionality to non-specific substances, an important area of research requiring further investigation (Jiang et al., 2018; Hu et al., 2019).

#### Efflux Transporters

Various efflux transporters are present at the BBB such as monocarboxylate transporter 1 (MCT1) for the efflux of lactic acid and small monocarboxylic acids, glucose transporter 1 (GLUT1) for the transport of glucose, etc. (Hladky and Barrand, 2018). Various ATP-Binding Cassette (ABC) transporters (such as P-glycoprotein, MRP2, and BCRP) (Cordon-Cardo et al., 1989; Sugawara et al., 1990; Hartz and Bauer, 2011) and solute carrier (SLC) transporters are also present at the BBB. The influx of chemotherapeutic neurotoxic agents (such as vincristine and doxorubicin) from blood to the brain parenchyma are prevented by the presence of P-glycoprotein (ABC transporter) at the luminal membrane of the BBB (Tatsuta et al., 1992; Virgintino et al., 2002; Chaves et al., 2014; Qosa et al., 2015; Hladky and Barrand, 2018). The absence of P-glycoprotein showed elevated drug levels (such as neurotoxic pesticide ivermectin, and carcinostatic drug vinblastine) in knockout mice brain (Schinkel et al., 1994; Geyer et al., 2009). SLC transporters move the waste products from the brain parenchyma to the endothelial cells, and then, either ABC or SLC transporters may drain these products further from the endothelial cells to the blood (Hladky and Barrand, 2018).

#### Transcytosis

Efflux by transcytosis includes adsorptive-mediated transcytosis (AMT) and receptor-mediated transcytosis (RMT) for the transport of large substrates across the BBB. However, there is little evidence for transcytosis in the direction of the brain parenchyma to the blood, although this parenchymal-blood transcytosis has been reported in studies involving the transport of Aβ peptides (Shibata et al., 2000; Bell et al., 2007; Qosa et al., 2014), IgG molecules (Zhang and Pardridge, 2001; Deane et al., 2005), insulin (Duffy and Pardridge, 1987), and transferrin (Banks et al., 1988) *via* the interaction with various receptor proteins.

Other non-specific substances such as albumin, inulin, sucrose, and dextran mainly exit the brain parenchyma *via* convective bulk flow through the perivascular spaces. For more details, refer to the thorough review article by Hladky and Barrand (2018).

## Waste Clearance External to the Brain Parenchyma

If waste products within the brain parenchyma are eligible to directly cross the BBB from the brain parenchyma to the blood, then they straightforwardly enter the highly specialized regional parenchymal venous circulation, which eventually drains into the dural venous sinuses, rejoining the internal jugular vein through the sigmoid sinus (Singh, 2009). However, if waste products enter the CSF compartments, several efflux routes participate in WC. Waste products that reach CSF from the brain parenchyma can be further taken out of the cranium *via* the proposed CSF outflow pathways: the arachnoid villi/granulations pathway and lymphatic pathways [which include peripheral lymphatic pathways *via* perineural spaces and meningeal lymphatic pathways] as indicated in Figure 1C.

### Arachnoid Villi/Granulations Pathway

Our classic textbook understanding of the role of the arachnoid villi/granulations in CSF transport comes from a study conducted over 100 years ago in the cadaver brain, under non-physiologically high CSF pressures (Weed, 1914). Our understanding is as follows: the CSF drains into the venous sinuses *via* the arachnoid villi/granulations and this is a major route of exit for the CSF as indicated in Figure 1Ci. However, recent studies have cast doubt on this understanding, inviting scientists to rethink the exact role and magnitude of the arachnoid villi/granulation route. Using high-resolution stereomicroscope and lymphatic-reporter mice, Ma et al. (2017) demonstrated that the lymphatic pathway, rather than the arachnoid villi/granulations, was the major outflow pathway for small and large molecular weight CSF tracers. Moreover, an MRI study conducted on normal and hydrocephalic rats, indicated that the superior sagittal sinus was not a significant draining channel for ventricular CSF tracers compared to the azygos internal cerebral vein, suggesting direct exit of the CSF tracer *via* the parenchymal venous circulation as opposed to exiting *via* the arachnoid villi/granulations (Krishnamurthy et al., 2018). To address the role of arachnoid granulations in the outflow of CSF, a recent study investigated the morphology of the arachnoid granulations in pigs utilizing electron microscopy and immunohistochemistry. They identified the cranial arachnoid granulation-like dural gap (CAG-LDG) in the meninges adjacent to the superior sagittal sinus however, they did not find CAG-LDG in the dura mater adjacent to the transverse sinus. They also demonstrated the lymphatic endothelial cell marker, Lyve-1 in the CAG-LDG in pigs, revealing characteristics similar to the lymphatic endothelium. This study provides a basis for further research of CSF drainage *via* these pathways and its relation to WC-associated diseases in humans (Kutomi and Takeda, 2020).

Nonetheless, historically, early studies of CSF transport from subarachnoid space to the vascular circulatory system suggested the presence of arachnoid villi/granulations located in the dura mater of the venous sinuses, providing an open unidirectional pathway connecting the meninges with the venous system (Welch and Pollay, 1961; Pollay, 2010). Arachnoid villi are solely depressions that are not fully penetrated into the dural wall before birth (Gomez et al., 1983; le Gros Clark, 1920). Arachnoid villi become functional and continue to grow with maturation and with an increase in CSF pressure (le Gros Clark, 1920). However, they start to degenerate and lose their efficiency with old age, which may result in the stagnation of CSF and impaired clearance of toxic waste products from the brain (Rubenstein, 1998). Perfusion studies through the arachnoid villi of African green monkeys suggested the diameter of arachnoid villi channels to be in the range of 4–12 μm (from histological data). Moreover, there was no discrimination in the passage between a variety of various sized particles as long as they were under the threshold value; which does not depend only on the diameter but also on the shape, the consistency as well as the surface properties associated with the particles (Welch and Pollay, 1961).

### Peripheral Lymphatic Pathways

It has been known for a long time that CSF drains *via* the perineural spaces of the cranial nerves, especially CNI in the nasal mucosa pathway for WC into the peripheral lymphatics outside the cranium; the nasal mucosa pathway acts as a major clearance route after intracisternal or intraventricular injections (Ma et al., 2017). However, several researchers have found drainage of CSF tracers along the perineural spaces of the other cranial nerves, complementary to the nasal mucosa pathway.

#### Nasal Mucosa Pathway

Many studies provide evidence for the nasal lymphatic pathway as the major pathway for CSF drainage, *via* channels in the cribriform plate of the ethmoid bone draining into the CLNs (Kida et al., 1993; Johnston et al., 2004) as indicated in Figure 1Cii. Macromolecular waste solutes such as Indian ink, albumin, Evans blue dye, etc. (Kida et al., 1993; Nagra et al., 2006), and immune cells such as GFP-expressing CD4 T lymphocytes, GFP monocytes, et cetera (Goldmann et al., 2006; Kaminski et al., 2012), all have been shown to exit the CNS through the nasal mucosa into the CLNs. Injection of radio-iodinated albumin into the caudate nucleus of rabbits demonstrated the drainage into deep cervical lymph nodes (dCLNs) majorly *via* the nasal submucosa, suggesting that the protein first passed through the cerebral perivenous spaces and then mainly entered the subarachnoid space of the ipsilateral olfactory lobe through the cribriform plate into the submucous space of the nose (Bradbury et al., 1981). Subsequent experiments in rabbits confirmed the major connection between CSF and dCLNs *via* the subarachnoid space prolongations around CNI into the nasal submucosa (Bradbury and Westrop, 1983); these experiments also showed that some solutes (depending on their molecular weight) leave the extracellular fluid in the nasal mucosa *via* peripheral capillary walls and drain into the blood. Similarly, the intraventricular injection of radiolabelled colloid in both rabbits and cats showed the passage of the tracer from nasal mucosa subsequently into the CLNs (Pile-Spellman et al., 1984). Kida et al. (1993) demonstrated that carbon particles reached into the lymphatics of the nasal submucosa *via* some discrete channels from the subarachnoid space of the olfactory bulb, following cisterna magna injection of Indian ink into the rats. Mollanji et al. (2001, 2002) also demonstrated the significance of the olfactory-lymphatic route over the arachnoid villi/granulations route, and represented it as a major drainage site for CSF transport by blocking the cribriform plate in sheep; indicating an increase in the resting intracranial pressure and a significant reduction in CSF clearance. Moreover, studies performed in sheep, pigs, rabbits, rats, mice, monkeys, and humans using injections of yellow Microfil into the CSF compartment identified the nasal lymphatic pathway as distinctive for CSF absorption in all mammals, showing the similar distribution of Microfil across all species (Johnston et al., 2004). More recent animal studies have also demonstrated similar findings and reached similar conclusions (Liu et al., 2012; Ma et al., 2017; Krishnamurthy et al., 2018). However, despite the crucial role of the nasal lymphatic pathway in the evacuation of molecular waste solutes from the CSF in various animal species, a recent *in vivo* MRI study in humans showed that, although CSF tracer (gadobutrol) was found near/below the cribriform plate, no significant amount of tracer reached the nasal mucosa itself, which questions the presence of this pathway in humans and warrants further research (Melin et al., 2020).

#### Other Perineural Spaces

CSF drainage from the subarachnoid space to the lymphatics outside the CNS is associated with the transport along cranial and spinal nerves (Brierley and Field, 1948; Zakharov et al., 2003; Krishnamurthy et al., 2018). Various studies have investigated the perineural routes along CNI (as discussed in the previous section) for CSF drainage into the CLNs, as the major CSF-lymphatic pathway (Kida et al., 1993; Johnston et al., 2004; Koh et al., 2005). Complementary to this nasal mucosa pathway, other studies have suggested the drainage of CSF tracer into extracranial peripheral lymphatics *via* perineural spaces of other cranial nerves. Arnold et al. (1972) showed the presence of contrast agent, radioisotopes in the perineural spaces of CNI, and the vestibulocochlear nerve (CNVIII) in guinea pigs a few minutes following CSF tracer injection, with most of the tracer ending up in dCLNs. Kida et al. (1993) observed the distribution of carbon particles along the perineural spaces of CNI, CNII, and the oculomotor nerve (CNIII) following the injection of Indian Ink into the cisterna magna of rats. Injection of radiolabeled albumin in the lateral ventricles of sheep also identified the perineural spaces along CNI, CNII, and CNVIII (Boulton et al., 1996). Moreover, Microfil infusion in the cisterna magna of neonatal sheep showed distribution along various cranial and spinal nerves; identifying the perineural routes along the trigeminal (CNV), the facial (CNVII), glossopharyngeal (CNIX), vagal (CNX), and hypoglossal (CNXII) nerves, with ultimate drainage into the extracranial lymphatics (Zakharov et al., 2003). Lee et al. (2015) demonstrated MRI CSF tracer (Gd-DTPA) distribution around/inside the cochlea (CNVIII) and pronounced efflux along CNX. Recently, Ma et al. (2017) found evidence of tracer exiting the perineural routes along CNI, CNII, and CNV in mice after intraventricular infusion. They also detected tracer exiting from the jugular foramina along CNIX, CNX, and accessory (CNXI) nerves, as well as from the stylomastoid foramina along CNVII, which ultimately drained to the dCLNs. Recently, Krishnamurthy et al. (2018) showed the distribution of MRI-labeled dextran along the spinal nerves, suggesting these nerves as the CSF outflow pathways from the spinal subarachnoid space to the lymphatics *via* perineural routes; as well as exit along the perineural spaces of cranial nerves (CNI, CNII, and CNV). In a previous study, we have demonstrated that all 12 pairs of cranial nerves and all 31 pairs of spinal nerves are surrounded with CSF in humans, which drain to regional peripheral lymphatics (Fahmy et al., 2021).

Although the utility of perineural spaces, peripheral lymphatic vessels, and CLNs in brain WC have been extensively studied, especially, after intraventricular or intracisternal injections, it remains unclear if perineural routes are the major routes for WC after the intraparenchymal injections (Ma et al., 2017). Moreover, it is not known if the waste solutes drain along the cranial nerves or actually enter the epineurium and then drain within the nerves along fascicles and/or axons (Benveniste et al., 2019). Therefore, further research is warranted in order to investigate the exact mechanism and role of the CSF-lymphatic pathway in brain WC.

### Meningeal Lymphatic Pathway

The presence of the meningeal lymphatic vessels was first introduced by Paolo Mascagni, an Italian physician at the end of the 18th century. He was an excellent anatomist, famous for his anatomical wax models with his scientific concern in lymphatic vessels in meninges. His work presented 28 wax models on the lymphatic system including three body-size models. However, his ideas on lymphatic vessels were not assessed for their value by contemporary researchers and forgotten (Lukić et al., 2003; Bucchieri et al., 2015; Da Mesquita et al., 2018a). In 1953, another Italian scientist inspected 30 human dura for lymphatic vessels and found them in only four dura (Lecco, 1953; Bucchieri et al., 2015). Later in 1996, Li et al. (1996) found the meningeal stomata in humans’ dura mater using a scanning electron microscope. However, they assumed the meningeal stomata to be a part of the prelymphatic capillary system, helping with cerebral lymph drainage. Mascagni’s observations were finally confirmed and presented in detail in 2015 when Louveau et al. (2015) identified the meningeal lymphatic vessels in mice lining the dural venous sinuses and demonstrated their ability to carry fluid and immune cells (such as T cells, MHCII^+^, CD11c^+^, and B220^+^, etc.) to the dCLNs. They also suggested that the meningeal lymphatic vessels were the primary route for the CSF-lymphatic pathway as compared to the nasal mucosa pathway. Also, the meningeal lymphatic vessels seemed to form a more complex and larger network along the transverse sinus, as compared to the superior sagittal sinus. They indicated that the transverse sinus meningeal lymphatic vessels may help in ISF and interstitial waste products clearance from the brain parenchyma, following their entry into the CSF compartment through the recently discovered glymphatic system pathway. At around the same time in 2015, Aspelund et al. demonstrated the presence of the meningeal lymphatic vessels in the dura mater of the CNS in mice and their exit *via* foramina at the base of the skull alongside the blood vasculature (arteries, veins) and cranial nerves (Aspelund et al., 2015) as indicated in Figure 1Ciii. Their data also suggested the clearance of ISF/CSF and interstitial waste products from the brain parenchyma to the dCLNs *via* meningeal lymphatic vessels after they traversed the glymphatic pathway. Another study on mice showed the meningeal lymphatic vessels as the drainage channel for macromolecules and immune cells in the CSF. They also revealed certain spots called “hot spots” along some meningeal lymphatic vessels which take up the tracer from CSF quickly after the injection as compared to other areas of the lymphatic vessels (Louveau et al., 2018). A recent study in mice showed the distinct morphological features of meningeal lymphatic vessels in the dorsal and basal dura along with their anatomical locations. They also demonstrated the basal meningeal lymphatic vessels as the hotspot for CSF macromolecular clearance and showed the impairment of meningeal lymphatic vessels and delayed CSF clearance with aging (Ahn et al., 2019).

Subsequently, the existence of the meningeal lymphatic vessels was reported using non-invasive, high-resolution clinical MRI in humans (Absinta et al., 2017; Naganawa et al., 2020) and non-human primates (Absinta et al., 2017). Another study in healthy human subjects showed the direction of lymphatic flow in meningeal lymphatic vessels to be opposite to the direction of venous flow in the superior sagittal sinus utilizing high-resolution MRI; suggesting it to reach the cribriform plate based on directionality (Kuo et al., 2018). Studies using confocal microscopy confirmed the presence of these meningeal lymphatic vessels in humans and indicated that, although these vessels served as potential efflux pathways for Aβ clearance from the brain, Aβ did not deposit in or around the meningeal lymphatic vessels (Goodman et al., 2018). Moreover, experiments in mice demonstrated that impairments to the meningeal lymphatic vessels were associated with decreased macromolecular influx and efflux in the perivascular spaces, which resulted in the accumulation of Aβ in the brain parenchyma (Da Mesquita et al., 2018b). This study also demonstrated a decrease in the diameter of meningeal lymphatic vessels in old mice and suggested the role of these vessels in AD pathology as well as in cognitive decline with aging. A recent human study identified the clearance of MRI contrast agent (gadodiamide) in putative meningeal lymphatic vessels, and the glymphatic system and suggested the impairment of both pathways with aging (Zhou et al., 2020). Another recent study in mice demonstrated that dorsal meningeal lymphatic vessels are critical for generating an effective immune response against brain tumors (Hu et al., 2020). Thus, the meningeal lymphatic vessels provide a route for draining macromolecules and immune cells from the CNS and therefore constitute a possible therapeutic target for the treatment of various neurodegenerative disorders.

## Conclusions and Future Directions

This review article discusses critical pathways for the removal of CSF/ISF and interstitial waste products from within and external to the brain parenchyma. It is a difficult task to classify these pathways by level of importance, as the scientific community remains unsure of the relative contribution of each of these pathways is to the overall process of brain WC. It may very well be that all these pathways must work in concert to ensure the effective and proper removal of toxic waste products from the brain and ensure healthy neuronal functioning. Further investigations are needed to synchronously assess the full picture of brain WC. Specifically, further investigations should include: (1) reexamination of fluid flow in the perivascular spaces as well as its directionality to resolve discrepancies between the glymphatic system and IPAD pathways; (2) evaluation of the role of AQP4 channels in perivascular spaces and brain parenchyma tracer distribution; (3) sophisticated modeling techniques including convective bulk flow coexisting with diffusion within the brain parenchyma to represent more realistic glymphatic flow; (4) reliable mathematical glymphatic system models using the periarterial space as elliptical instead of the circular annulus; (5) determination of the exact location of the meningeal lymphatic vessels within the meningeal layers in various species and the mechanism of CSF exit into these vessels; (6) develop directionality/causation to the association between glymphatic dysfunction and neurological diseases, in order to develop clinical therapeutics; (7) reexamination of the arachnoid villi/granulations for their possible role in CSF outflow; and (8) *in vivo* reexamination of the nasal lymphatic pathway *via* the cribriform plate in the humans.

## Author Contributions

JK, LF, and QJ have made substantial contribution to the concept, design, and drafting of the manuscript. JK, LF, QJ, MC, ZZ, ED-B, LZ, JH, and GD critically revised the article, provided feedback, and helped in shaping the manuscript. JK, LF, QJ, ZZ, and MC critically reviewed to approve the final version to be published. All authors agree to the published version of the manuscript. All authors contributed to the article and approved the submitted version.

## Conflict of Interest

The authors declare that the research was conducted in the absence of any commercial or financial relationships that could be construed as a potential conflict of interest.
